# The Impact of Integrated Community Case Management of Childhood Diseases Interventions to Prevent Malaria Fever in Children Less than Five Years Old in Bauchi State of Nigeria

**DOI:** 10.1371/journal.pone.0148586

**Published:** 2016-02-04

**Authors:** Dele Abegunde, Nosa Orobaton, Amos Bassi, Olugbenga Oguntunde, Moyosola Bamidele, Masduq Abdulkrim, Ezenwa Nwizugbe

**Affiliations:** 1 John Snow Incorporated (JSI) Research and Training Institute, Boston, Massachusetts, United States of America; 2 Futures Group, Chapel Hill, North Carolina, United States of America; University College London, UNITED KINGDOM

## Abstract

**Background:**

Malaria accounts for about 300,000 childhood deaths and 30% of under-five year old mortality in Nigeria annually. We assessed the impact of intervention strategies that integrated Patent Medicines Vendors into community case management of childhood-diseases, improved access to artemisinin combination therapy (ACT) and distributed bed nets to households. We explored the influence of household socioeconomic characteristics on the impact of the interventions on fever in the under-five year olds in Bauchi State Nigeria.

**Methods:**

A cross-sectional case-controlled, interventional study, which sampled 3077 and 2737 under-5 year olds from 1,588 and 1601 households in pre- and post-intervention periods respectively, was conducted from 2013 to 2015. Difference-in-differences and logistic regression analyses were performed to estimate the impact attributable to the interventions: integrated community case management of childhood illness which introduced trained public and private sector health providers and the possession of nets on the prevalence of fever.

**Results:**

Two-week prevalence of fever among under-fives declined from 56.6% at pre-intervention to 42.5% at post-intervention. Fever-prevention fraction attributable to nets was statistically significant (OR = 0.217, 95% CI: 0.08–0.33). Children in the intervention group had significantly fewer incidence of fever than children in the control group had (OR = 0.765, 95% CI: 0.67–0.87). Although being in the intervention group significantly provided 23.5% protection against fever (95% CI: 0.13–0.33), the post-intervention likelihood of fever was also significantly less than at pre-intervention (OR = 0.57, 95% CI: 0.50–0.65). The intervention protection fraction against fever was statistically significant at 43.4% (OR = 0.434, 95% CI: 0.36–0.50). Logistic regression showed that the odds of fever were lower in households with nets (OR = 0.72, 95% CI: 0.60–0.88), among children whose mothers had higher education, in the post-intervention period (OR = 0.39, 95% CI: 0.33–0.46) and in the intervention group (OR = 0.52, 95% CI: 0.48–0.66). The odds of fever increased with higher socio-economic status of households (17.9%-19.5%). Difference-in-differences showed that the interventions significantly reduced occurrence of fever in the intervention group (OR = 1.70, 95% CI: 1.36–2.14).

**Conclusion:**

The interventions were effective in reducing the prevalence and the likelihood of childhood malaria fever. Taken to scale, these can significantly reduce the burden of malaria fever in the under-five year old children.

## Background

Malaria remains a major cause of morbidity and mortality among children less than five year old particularly in Sub-Saharan Africa despite several decades of exposure to malaria control interventions. In 2012, The World Health Organization (WHO) estimated that 90% of all malaria deaths worldwide occurred in the Africa region and mostly among children under-five years of age (under-fives). Although malaria-specific mortality rates among children in the region have declined by an estimated 54% since 2000, high endemicity persists in many Sub-Sahara African countries including Nigeria [1] where it accounts for about 110 million clinically diagnosed cases per year, an estimated 60% of annual outpatient visits and 30% of all hospitalizations., About 300,000 children in Nigeria die from malaria annually [1]. About 25% of infant mortality and 30% of mortality in children under-five years in Nigeria are attributed to malaria [1].

Although malaria has been eliminated in Europe, Asia and the Americas, efforts to eradicate malaria in Sub-Saharan Africa have largely been less than successful [2]. Regional and international efforts to fight malaria since the 1940s have recently been subsumed into a coordinated global response. In 1998, WHO, UNICEF, United Nations Development Program (UNDP) and the World Bank launched the Roll Back Malaria (RBM) partnership to achieve universal coverage of malaria control interventions. The Millennium Development Goals (MDG) Malaria summit in 2008 endorsed a Global Malaria Action Plan aimed at achieving a substantial reduction in the burden (elimination in 8–10 countries including Nigeria, and the eventual eradication) of malaria [3]. Since its inception in 2002, the Global Fund to Fight AIDS, Tuberculosis and Malaria established as an international financing mechanism to fight malaria (including AIDS and Tuberculosis), have thus far committed over US$17billion in funds to about 144 countries and have supported the distribution of 122 million bed nets and financed the delivery of over 142 million drug treatments encounters to fight malaria. In addition, it has committed over $ 8.33 billion to malaria-related initiatives in 80 countries [4]. The Bill and Melinda Gates Foundation has supported the development of new tools to combat malaria through innovations and scientific research [5].

Malaria has exacted severe socio-economic burden on countries and malaria endemic communities. In Nigeria, an estimated 132 billion Naira (2013 USD 825 million)—in treatment costs, prevention, loss of work time—is lost to malaria annually [6]. Nigeria developed a comprehensive National Malaria Control Strategic Plan (NMCSP) to “reduce deaths and prevalence of malaria in children under-five years old; increase ownership and use of insecticide-treated nets (ITNs) and long-lasting insecticidal nets (LLINs); increase the use of diagnostic tests; and improve appropriate and timely treatment of malaria; among other strategies”[6]. Millions of ITNs and LLIN have so far been distributed in Nigeria. According to the Nigerian Demographic and Health Survey (NDHS) 2013, the percentage of households in Nigeria with at least one of any type of mosquito net rose from 17% in 2008 to 55% in 2013[1]. Similarly, during the same period, households with at least one insecticide-treated net (ITN) rose from 8% to 50%. Household possession of at least one bed net was higher in rural households (61%) than in urban households (48%) [1]. Inspired by the Millennium Development Goals (MDGs), international partners notched up support to national programs in the distribution of ITNs, particularly to rural Nigerian communities.

Despite the development of these proven, preventive interventions to reduce the burden of malaria and the availability of effective treatment technologies, access to effective management of malaria remains poor in Nigeria. Evidence has shown that community case management (CCM) of malaria by appropriately trained, well-supervised community health workers supported with uninterrupted supply of medicines, can help reduce overall and malaria-specific under-five mortality by between 40 and 60 per cent, respectively, and severe malaria morbidity by 53%. [7–9] Unicef, WHO and partners have actively supported integrated community case management strategy in an increasing number of countries to train, supply and supervise front-line providers to treat childhood malaria in malaria-affected countries using artemisinin-based combination therapy (ACT). In addition, community level testing for malaria has been facilitated by the availability of high-quality rapid diagnostic tests (RDTs) for malaria. [9]

### Intervention

A John Snow Inc. (JSI) managed United States Agency for International Development (USAID) flagship health program between 2009 to 2015, supported state-led programs that strengthened maternal, newborn and child health interventions, including the upgrade of health facilities for more optimal provision of treatment for malaria. The Bauchi State Agency for Tuberculosis and Malaria Control (BACATMA), funded in part by the World Bank Malaria Booster program, adopted a strategy that incorporated Patent Medicines Vendors (PMVs) as care providers in the treatment of malaria. Between May and August 2014, about 188 PMVs and 206 service providers (SPs) drawn from public health facilities across two intervention (Tafawa-Balewa and Ganjuwa) local government areas (LGAs) in the State received training based on an integrated community case management of childhood diseases (iCCM) modular toolkit and implementation guide for community case management of childhood illnesses [10]. In addition to these interventions, the trained PMVs and SPs obtained supportive supervision on the field between August and September 2014. Training of PMVs, SPs and supportive supervision were not carried out in the two control (Alkaleri and Ningi) LGAs. The intervention also included the improvement of the supply of commodities, the provision of Artemisinin-based Combination Therapies (ACTs) and rapid diagnostic tests (RDTs) to public facilities.

The state had distributed 1.8 million nets to households in all LGAs in March 2010 before these interventions began in 2013. Another household distribution of 3.2 million ITNs was repeated between October and November 2014 in all LGAs. The 2010 bed net rollout distributed an average of 2.7 and 0.6 bed nets per households and per capita respectively, in every Ward of every LGA in the state. These two rounds of bed net distribution roughly coincided with the beginning and the end of the intervention program, providing opportunity to examine the effects of bed net distribution 5 years apart, on childhood malaria. In the first year of the intervention project starting in August 2013 and after a baseline survey was completed in November 2013, skills capacity of point of service (POS) in the intervention LGAs to deliver appropriate integrated community case management of malaria was improved through training [11]. The service provision component of the interventions occurred between May 2014 and October 2014.

### Objectives

The purpose of this study was to estimate the impact of the interventions that wereimplemented to mitigate morbidity due to malaria fever and to explore the association between household characteristics including the possession of insecticide treated bed nets (ITN and LLIN) and the prevalence of malaria fever in children under five years old. In this respect, we posit a batch of hypotheses. The first batch includes a null hypothesis that household possession of bed nets did not necessarily influence the household prevalence of malaria fever in which case the odds ratio of one (the null hypothesis) will not be rejected and the prevention fraction of household possession of bed nests will be zero. The alternate hypothesis was that households that possessed ITN experienced less malaria morbidity (measured as fever, with high index of suspicion for malaria). In this case, the odds ratio of one and the prevention fraction of household possession of bed nets of zero were rejected; (2) The second batch includes the hypothesis was that the interventions did not affect the morbidity of malaria fever in under-five year old children and the alternative hypothesis was that interventions affected the malaria morbidity.

### Study Location

Bauchi State is located in the northeastern zone of Nigeria with an estimated population of 5.9 million in 2015 inhabitants as projected from the 2006 national population census; 1.14 million were children under the age of five years. In 2013 the Nigeria Demographic and Health Survey (NDHS) estimated that 25.3% of under-five year old children in Bauchi State were reported to have had fever in the two weeks preceding the interview, which was double the national prevalence of 12.5% [1]. Malaria transmission is high all year round especially after the end of the raining season [1].

## Methods

A case controlled, pre- and post-intervention study was design to evaluate the integration of Patent Medicine Vendors (PMVs) into integrated community case management (iCCM) of childhood diseases including malaria. Using a multi-stage stratified cluster design, data was obtained data from 3,910 sampled households: 2277 and 1633 households in the pre- (baseline survey was conducted in November 2013) and post-intervention periods (end-line survey was conducted in January 2014) respectively, across the two control (Alkaleri, Ningi) and two intervention (Tafawa-Balewa, Ganjuwa) local government areas (LGAs). The control LGAs and the intervention LGAs are located in the northwest and southeast regions of the State. They were selected purposively to avoid shared boundaries to minimize spillover effects and for their accessibility to Bauchi City, the capital city. Each. These LGAs are located on the east, west, south and central geo-political regions of the state and were selected for the study for their accessibility from the central administrative region. Random samples of cluster (national census enumeration areas) were obtained from each of the four LGAs. Samples of household were obtained from the clusters through systematic randomization. All the under-five year olds in each household were included in the study. Using a pretested questionnaire administered on the mothers (caregivers), the study captured data on household socio-demographic characteristics of the children and their caregivers including the management of malaria, the number, types and duration of possession of the bed nets. The history of fever was used as a proxy for malaria fever similarly to the NDHS 2013 [1]. Caregivers were asked if any child under five year old in the household had had fever, diarrhea or cough in two weeks preceding the interview. They were then asked where they had sought care and what treatments including medications they were given. Data from 5,814 under-five children from sampled households—3077 and 2737 in the pre-and post-intervention periods—were obtained for the study.

### Ethics Statement

Ethical approval for the study was obtained from the Bauchi State Ethics Review Board. Written, informed consent was obtained from heads of households and mothers (caregivers) after each one had received a detailed explanation of the purpose of the study and their rights to freely, decline participation without any reservations whatsoever, at any time in the survey. Confidentiality policy was explained to all respondents.

### Analysis

Contingency table analysis was used to partition the cases with fever from non-fever cases classified by exposure or non-exposure to bed nets, to estimate and compare the odds ratio of malaria fever in children who lived in households with insecticide treated bed nets with those who lived in households without insecticide treated bed nets. The odds ratios of fever in the pre- and post-intervention periods, in the intervention and control LGAs were also estimated. In addition, fever prevention fraction attributable to bed nets usage, and the interventions were estimated to demonstrate the impact of the interventions. We implemented a logistic regression model (Eq 1), and used fever/no-fever *(F*_*it*_*)* as the outcome variable, at study group *i* (control LGA = 0, intervention LGAs = 1) and period *t* (pre-intervention = 0, post-intervention = 1) against the study-group *(I*_*it*_*)* and period (P_*it*_) dummies including interaction between study-group and period ((P*I)_*it*_). This interaction term is an estimator of the prevention (or attributable) fraction and the overall impact of the interventions on reduction of the prevalence of fever. This was essentially a difference-in-differences analysis to adjust the impact of the interventions for the changes that may have occurred unprovoked in the control group between the pre- and post-intervention periods. The estimates derived from the logistic model improved on the contingency table estimation in that the logistic regression allowed for further stratification of the explanatory variables, and controlled for the confounding effects *(C*_*it*_*)*.

$$F_{hi}=\;\beta_{1}P_{t}+\;\beta_{2}I_{i}+\beta_{3}{\left(P*I\right)}_{it}+C_{it}\;\varepsilon_{it}$$ (1)

The age and educational background of the mothers (caregivers), the number of children under-five years who lived in a household [12, 13] and the socio-economic background [14] of households can influence household demand for, possession and use of bed nets on the one hand [14, 15] and the risk of contracting malaria on the other [15]. For instance, economically advantaged households may have had better access to bed nets and improved housing conditions that reduced the risk of parasite transmission than the less economically advantaged households. We used Principal Component Analysis (PCA) to construct an index of household socioeconomic wealth from household durable assets [16–18]. The wealth index scores were then used to categorize households into to low-, middle- and high-socio-economic status included as an independent variable in the analysis. The logistic regression model was weighted with inverse probabilistic sample weights to account for differential probabilities in each sample because of the nature of the multistage cluster design and to improve the accuracy of the estimates. Analyses were performed with STATA^(C)^ version 12; statistical level of confidence was set at 95%. Bayesian and Akaike’s Information Criteria (BIC & AIC), McFadden’s R^2^ were used to select the best-fit and best-specified logistic model. The variables of the logistic regression are described in Table 1.

**Table 1:** Description of variables.

	Pre-intervention			Post- intervention		
Variable description	Control group	Intervention group	Pre-intervention Total	Control group	Intervention group	Post intervention Total
**Total**	1,120	1,157	2,277	730	903	1,633
**Fever**	676 (60.4%)	613 (53.0%)	1,289 (56.6%)	327 (44.9%)	366 (40.6%)	693 (42.5%)
**No Fever (base case)**	444 (39.6%)	544 (47.0%)	988 (43.4%)	402 (55.1%)	536 (59.4%)	938 (57.5%)
**Bed net in household**	815 (72.8%)	849 (73.4%)	1,664 (73.1%)	554 (75.9%)	869 (96.2%)	1,423 (87.1%)
**No bed net (base case)**	305 (27.2%)	308 (26.6%)	613 (26.9%)	176 (24.1%)	34 (3.8%)	210 (12.9%)
**Mean age of caregiver (inyears)**	27.0	27.8	27.3	26.8	28.2	27.5
**Socioeconomic Status:**						
Low (base case)	337 (30.1%)	324 (28.0%)	661 (29.0%)	367 (50.3%)	371 (41.1%)	738 (45.2%)
Middle	230 (20.5%)	256 (22.1%)	486 (21.3%)	100 (13.7%)	117 (13.0%)	217 (13.3%)
High	553 (49.4%)	577 (49.9%)	1,130 (49.6%)	263 (36.0%)	415 (46.0%)	678 (41.5%)
**Caregiver educational level:**						
None (base case)	1,001(89.4%)	796 (68.8%)	1,797 (78.9%)	649 (88.9%)	569 (63.0%)	1,218(74.6%)
Primary	75 (6.7%)	255 (22.0%)	330 (14.5%)	6 (9.0%)6	237 (26.2%)	303 (18.6%)
Secondary	41 (3.7%)	93 (8.0%)	13 (5.9%)4	15 (2.1%)	89 (9.9%)	104 (6.4%)
Higher	3 (0.3%)	13 (1.1%)	16 (0.7%)	0 (0.0%)	8 (0.9%)	8 (0.5%)
**Mean age child (in years)**	2.6	2.3	2.5	2.2	2.3	2.2

## Results

Households with bed nets increased from 73.1% in the pre-intervention period to 87.1% post-intervention, and attained as high as 96.2% in intervention households, (Table 1). The estimated two-week occurrence of fever among under-fives declined from 56.6% at pre-intervention to 42.5% in the post-intervention period. Fever, however, was more prevalent in the control group than in the intervention group in both the pre- and post-intervention periods. The mean age of the mothers (caregivers) was comparable (about 27 to 28 years) across the study groups. Majority of the caregivers (78.9% and 74.6% in the pre- and post-intervention periods respectively) had no formal education. The mean age of the sampled children ranged from 2.2 to 2.6 among the study groups.

As shown in the contingency table (Table 2), the odd ratio (OR) of fever among the under-fives in households with bed nets relative to those without, was 0.783 [Confidence Interval (CI):0.67–0.95]. As such, the null hypothesis that the odds ratio is one was rejected; this implied that children who lived in households that possessed ITNs reported the incidence of fever 21.7% fewer times than children who lived in households without ITNs. The fever prevention fraction attributable to possession of bed nets in the household was statistically significant at OR = 0.217 (95% CI: 0.08–0.33). Similarly, children in the intervention group were significantly less likely to have malaria fever compared to children in the control group (OR = 0.765, 95% CI: 0.67–0.87) at the end of the study. The intervention prevention fraction was statistically significant at OR = 0.235 (95% CI: 0.13–0.33). The likelihood of fever in children post-intervention was also significantly less than the pre-intervention period (OR = 0.566, 95% CI: 0.50–0.65). The entire intervention strategy that was implemented significantly protected against malaria fever, (Prevention Fraction was OR = 0.434, 95% CI: 0.36–0.50).

**Table 2:** Contingency Table of the Effect of Household Possession of Bed Nets, on the Risk of Fever in Children.

Category	No. with Fever	No. with no Fever	Total	Proportion with fever	Odds Ratio (95% Confidence Interval)	Prevention Fraction. (95% Confidence Interval)	Statistic
**Bed nets**	1525	1560	3085	49.4%			
**No Bed nets**	457	366	823	55.6%	0.783	0.217	
**Total**	1982	1926	3908	50.72%	(0.67–0.92)	(0.08–0.33)	Pr>chi2 = 0.0
**Intervention**	979	1080	2059	47.6%			
**Control group**	1003	846	1849	54.4%	0.765	0.235	
**Total**	1982	1926	3908	50.72%	(0.67–0.87)	(0.13–0.33)	Pr>chi2 = 0.0
**Post-intervention**	693	938	1631	42.5%			
**Pre-intervention**	1289	988	2277	56.5%	0.566	0.434	
**Total**	1982	1926	3908	50.72%	(0.50–0.65)	(0.36–0.50)	Pr>chi2 = 0.0

The logistic regression in Table 3 showed that the estimated odds ratio of fever in children under-fives in households with ITNs and those in households without was 0.72 (95% CI: 0.60–0.88), an improvement from the estimated OR of 0.783 from the contingency analysis in Table 2. This indicates the odds of fever in under-fives in households with ITNs were significantly lower than the odds of fever in households without ITN and that bed nets offered 27.8% protection against malaria fever. The odds of fever in the under-five years old increased with the age of the mothers (or caregivers) (OR = 1.005, 95% CI: 0.997–1.01) although this was not significant. The odds ratio for households in the middle- and high- socio-economic levels relative to households in the low socioeconomic level, were (OR = 1.195, 95% CI: 1.03–1.39) and (OR = 1.179, 95% CI: 1.02–1.36) respectively. As such, the odds of fever in the under-five year olds that lived in middle and high socio-economic households were significantly higher than that of fever in low socio-economic households. The results also found that the educational levels of mothers (caregivers) influenced the odds of fever in under-five year olds. The odds of fever were lower with higher levels of mother’s education. The odds of fever in under-five year olds whose mothers had primary education did not differ significantly from those without formal education (OR = 1.007, 95% CI: 0.85–1.19). The odds of fever in the under-five year olds whose mothers had secondary education (OR = 0.69; 95% CI: 0.54–0.87) and tertiary education (OR = 0.45; 95% CI: 0.22–0.91) were significantly lower than for children of mothers with no formal education. There was a slight but significant decrease in the odds of fever as the number of the under five-year olds resident in households increased (OR = 0.995, 95% CI: 0.99–0.997).

**Table 3:** Results of the Logistic Regression of Childhood Fever on Household Possession of Bed Nets, and Household Characteristics.

Fever	Odds Ratio	Standard Error.	z	P>z	95% Confidence Interval	
**Household net possession**	0.722	0.071	-3.290	0.001	0.595	0.877
**Caregiver’s age**	1.005	0.004	1.170	0.241	0.997	1.013
**Socioeconomic level:**						
Middle	1.195	0.090	2.350	0.019	1.030	1.386
Highest	1.179	0.087	2.240	0.025	1.021	1.362
**Mother’s educational level:**						
Primary education	1.007	0.085	0.090	0.931	0.854	1.189
Secondary education	0.688	0.083	-3.100	0.002	0.544	0.872
Tertiary education	0.449	0.161	-2.240	0.025	0.222	0.906
Number of under-5 in household	0.995	0.001	-5.220	0.000	0.994	0.997
**Time period (base = baseline)**	0.390	0.032	-11.310	0.000	0.332	0.460
**Treatment (base = control)**	0.562	0.046	-7.090	0.000	0.480	0.659
Interaction: Treatment*Time period	1.703	0.198	4.590	0.000	1.357	2.138
**_cons**	2.269	0.380	4.900	0.000	1.635	3.150

Log Pseudolikelihood = -1089646, Wald chi^2^ (11) = 205.9, Probability > chi2 = 0.0000, Pseudo R^2^ = 0.34

The likelihood of fever in under five-year olds was significantly lower at post-intervention than at pre-intervention (OR = 0.39, 95% CI: 0.33–0.46) and this was the case with the treatment group compared to the control group (OR = 0.56, 95% CI: 0.48–0.66). The interaction between the treatment and period variable was also significant (OR = 1.703, 95% CI: 1.64–3.15).

## Discussion

This study primarily estimated the impact of the interventions implemented to mitigate morbidity from malaria fever and explore how household characteristics influenced the impact of the interventions for reducing the prevalence of fever in the under five year olds in Bauchi State of Nigeria. These interventions consisted of two rounds of bed net distribution to households in all LGAs in the state on the one hand, and the integration and training of PMV into integrated management of childhood diseases including malaria, increased access to ACTs and training of service providers (through iCCM implementation) on the other. The results showed that household bed net coverage increased on average from 73.1% in the pre-intervention period to 87.1% post-intervention. Coverage reached 96.2% in households in the intervention groups at the end of the intervention period. The pre-intervention bed net coverage compared well with a previous coverage of 87% estimated in Bauchi State [15] in 2011. It was not clear why the intervention areas had better household bed net coverage, since all LGAs had a uniform average of 2.7 bed nets per household in 2015. A possible explanation suggesting that bed nets distribution in the first round could have been less in the control LGAs is farfetched. A study in Nigeria showed differences in net survival rates across regions [19]. Differing net survival rates between the study-groups may account for the differences in the coverage of bed nets found in this study. A more plausible explanation however, is that the observed differences in household coverage of bed nets was likely spin-offs from the activities of the trained Chemist, PMVs and SPs within health centers in the intervention areas as a consequence of the study.

The results showed that the two-week occurrence of fever among under-fives declined from 56.6% pre-intervention, to 42.5% post-intervention although the observed high prevalence of fever in the control group compared to the intervention group in both the pre- and post-intervention periods was statistically significant. Children that lived in households that possessed ITNs reported 21.7% less fever than children who lived in households without ITNs. Children in the intervention group were significantly less likely to have malaria fever relative to children in the control group, at the post intervention period. Being in the intervention group of the study significantly conferred on children, as much as 23.5% prevention against fever. The likelihood of fever at post-intervention was also significantly less than the pre-intervention period and protection fraction against fever attributable to the intervention, was statistically significant at 43.4%

The logistic regression showed the estimated adjusted odds ratio of fever in children under five years old in households with ITNs and those in households without was 0.722. Household possession of bed nets therefore provided about 27.8% protection against fever. The age of mothers (or caregivers) did not significantly predict the odds of fever in the under-five. The odds of fever in the under-five year olds living in the higher and the highest socio-economic households were 19.5%, 17.9% higher than the odds of fever in low socio-economic households. The results also showed that the educational levels of mothers (caregivers) influenced the odds of fever in the under-five year olds. Higher level of mother’s education significantly protected the children against fever. The odds of fever significantly decreased slightly with the number of the under five-year olds resident in households implying that children in households with high number of under-five year olds were slightly less likely to report fever than households with fewer children. Logistic regression also showed that the intervention significantly reduced occurrence of fever.

With regards to the influence of household demographic characteristics, on net ownership and use—which in this study, showed positive association between higher levels of mother’s education and high net ownership—a previous study in Bauchi State in 2011 similarly reported that mothers’ education and higher socioeconomic factors were positively associated with possession and use of bed nets [15]. Another study in Angola showed that children from wealthier households were less likely to be parasitaemic than those in poorest households. The poorest households in Tanzania, and Uganda were less likely to own a net. However, the poorest household in Angola had higher usage of bed nets than children in wealthier households [14]. In this present study, compared with mothers with no education, mothers’ education significantly protected against fever by a factor of as much as 31.2% in the children of mothers with secondary and by and 55.1% among children whose mothers had tertiary education respectively. Although the likelihood of fever was the same with the low and middle socioeconomic levels, the children in the highest socioeconomic group had 16.7% increased odds of fever relative to the low socioeconomic households. Households with higher socioeconomic profiles by implication have better economic access to factors of fever prevention and therefore, should experience lower prevalence rates of fever. Possible interactions between household possession of ITNs and the other unobserved factors may have contributed to this contrarian outcome. For instance, the Nigerian DHS of 2013 showed that possession of ITNs was more (61%) in rural households than in urban households (48%) [1]. Rural households however, were arguably less likely to be as socio-economically endowed as urban households were. So also, mothers with secondary and tertiary education are likely concentrated in the urban areas. The higher possession of ITNs in rural households despite lower household income relative urban households and a higher concentration of women with low to no education, largely reflect the subsidy policy of of Bauchi State government that delivered free ITNs targeting the poor and rural communities. It is likely that the subsidy program introduced market distortions in the possession of ITNs that obviated the expected positive relationship between socioeconomic status (household wealth index) and possession of ITN. In this respect, it can argued that the lack of association between household wealth and net ownership is an indication of the impact of the free bed net distribution in the mitigation of inequalities in household bed net ownership and in childhood malaria fever.

Overall, the interventions implemented in this study which included bed nets distribution and the integration of PMVs into integrated management of childhood malaria, significantly decreased the odds and significantly protected the children against fever. Increased bed nets coverage alone provided a significant 21.7% protection against fever whereas; the collective intervention provided as much as 58.1% protection. The impact recorded in this study demonstrate the potential in innovative public-private partnership for the reduction of common childhood diseases particularly malaria fever which is endemic in Bauchi state. The results underscore the evidence that operations of the Chemists and PMVs (including that of SPs) can be streamlined into providing integrated community case management of these childhood diseases particularly in malaria endemic communities where poor health systems and access to appropriate treatment are limited.

### Study Limitations

There were some limitations to this study. The use of mothers’ (caregivers’) account of fever in the under-fiver year olds as proxy for malaria may likely have compromised the accuracy of the outcome variable relative to using more confirmatory but invasive techniques that measure parasitic load such as; the Entomological Inoculation Rate (EIR) [20], the relatively new RDT and blood smears [21]. Invasive options would have potentially increased the cost and timeliness of the study, in addition to the potential ethical challenges. However, malaria is often clinically diagnosed and treated with reasonably high index of suspicion at the primary care level in endemic countries like Nigeria–although over-diagnosis and treatment commonly occur. The proportion of cases of fever in the under five year old children treated as malaria which were slide-positive varied from 16.9% in a study in Lagos Nigeria [22], to 68.8% in a study from Tanzania [23]. Studies such as the waves of the Nigerian Demographic Health Surveys have conveniently used the same indicator for malaria fever as was employed in this study [1]. Since there was no evidence that the possible measurement bias was selective on either the study or control groups, the effect of this approximation on the outcome of this study was therefore likely to be minimal. The study coincided with the peak of transmission although transmission is all year round. The outcomes of this study may vary if the temporal variation in transmission was accounted for. Nevertheless, the results are sufficiently robust to inform future strategies to eliminate malaria, particularly in Nigerian populations.

## Conclusion

We conclude that interventions that concurrently combine bed nets distribution, with public-private partnerships with chemists and patent medicine vendors given appropriate training on treatment of fevers, training of SPs in health facilities, enhanced supplies and distribution of commodities, can be effective for reducing the prevalence and the likelihood of fever occurring in children under five years. Taken to scale, these combination of strategies can contribute significantly to the reduction of reduce the burden of malaria fever in the under-five year old children in Nigeria.
